# History of Neurotrauma in Ancient Greece

**DOI:** 10.25122/jml-2022-1003

**Published:** 2022-03

**Authors:** Stefana-Andrada Dobran, Livia Livint Popa, Dafin Muresanu

**Affiliations:** 1. RoNeuro Institute for Neurological Research and Diagnostic, Cluj-Napoca, Romania; 2. Department of Neuroscience, Iuliu Hatieganu University of Medicine and Pharmacy, Cluj-Napoca, Romania

The history of neurotrauma in Ancient Greece intertwines science with literature in an awe-inspiring and fascinating approach. There is great importance in understanding the historical context of medical and scientific progress, as it highlights excellent insight into the epidemiology, causes, and treatments of neurotraumatology. At the same time, it offers a fascinating glimpse into the world of the past. By presenting the stories and achievements of the great minds that have come before us, we celebrate their work and introduce people of all backgrounds to the history and evolution of neurotrauma.

In ancient times, illness was believed to be cured and caused by the gods (ex: epilepsy, hysteria, insanity – known as "miasma" in the times of Homer) [1]. Nonetheless, ancient Greeks possessed significant knowledge on the anatomy of the head and neck and the pathophysiology of neurotrauma, holding insight on the results of severe trauma (*e.g.*, quadriplegia, loss of consciousness) [2].

The Greeks were farmers, athletes, and warriors, with military trauma accounting for as high as 93.68% of all traumas [3]. A fascinating trait of ancient Greek civilization is that they might be one of the first civilizations to recognize the brain as the center of consciousness or spirit (psyche). In contrast, the ancient Egyptians, Mesopotamians, and Hebrews often regarded the heart as the central organ [1]. Another inspiring observation is that ancient Greeks might have been aware of the association between pupillary dilation and severe brain injury or loss of consciousness due to the frequent use of the phrase "darkness enfolded his eyes" used when describing cases of neurotrauma [1].

The most relevant observations in ancient Greece were in the works of Hippocrates, Celsus, and Galen, who mentioned changes in consciousness following traumatic brain injury (TBI) and significantly contributed to the advancement of medical sciences through the development of treatment methods [4]. To illustrate this concept, it is worth mentioning that the works of Hippocrates include classifications, descriptions, and suggestions for the approach of various types of fractures. Furthermore, Celsus described the management of depressed fractures, while Galen focused on describing new tools and methods for perfecting surgical procedures [4].

**Figure 1.**
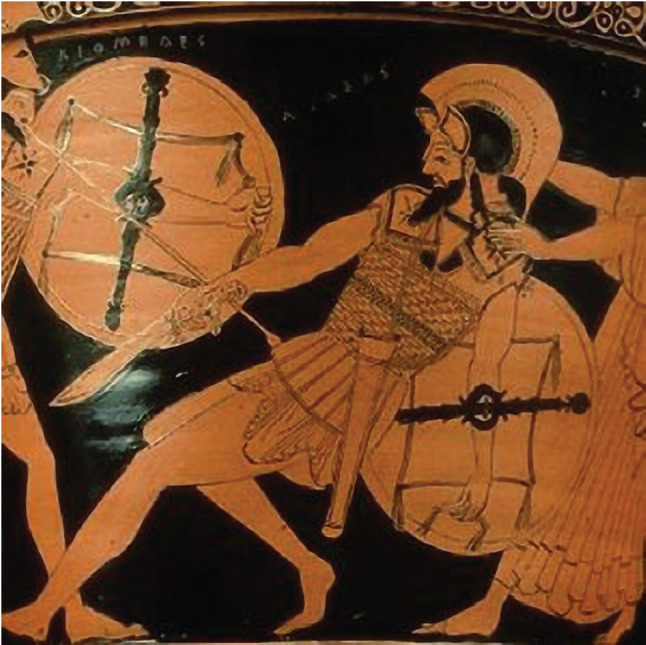
Cranio-maxillofacial injuries in Homer's Iliad [6].

The Homeric Period (XI-VIII BC), named after the famous Greek poet, revealed some of the first sources of Greek knowledge on neurotrauma in the great literature classics, the Iliad and the Odyssey [1, 2, 5]. The poems include descriptions of injuries due to skull indentation (Odyssey) and descriptions and comparisons of penetrating wounds resulting from spears, swords, and arrows (Illiad) [5]. Homer describes 41 injuries of the head and spine in his works, with a vast majority being fatal [2]. It could be speculated that the well-known poet was also active as a wartime surgeon due to the extensive details on trauma presented in his works (Figure 1) [1, 6].

Homer, Plato and Plutarch, along with other minds of the time, depict, in their writings, cranial trauma on the battlefields, in accidents, or during athletic games, such as chariot races and boxing (most traumatic sport) [2]. A fascinating case described by Plutarch was the death of king Pyrrhos, who suffered a concussion after being hit in the head with a tile by the mother of an opposing warrior hurt in battle, resulting in him falling off the horse and being killed. However, the irony of the story is that the battle was won by Pyrrhos's army, leading to the birth of the term "Pyrrhic victory", which describes a victory obtained at a great cost [2].

In ancient Greece, those affected by neurotrauma were primarily men, and the consequences mentioned included "loss of consciousness, quadriplegia, brain, and brain stem injuries", with trauma being directed mainly towards the faces and necks, as the heads were often protected by helmets [2].

Some of the most notable mentions of trauma were those written by the poet Aeschylos, king Pyrrhos, and Alexander the Great. In their works, they offer insight into the causes of neurotrauma, as they describe the trauma caused by the most lethal weapons of the time: spear (58% of cases), sword (21%), and stones (15%) [2]. The injuries were described in great detail, as it can be seen in the mention of the lethal damage caused by a spear of Antilochos of Echepolos: "Him [Echepolos] was he first to strike on the ridge of his helmet with crest of horsehair, and into his forehead drove the spear, and the spearpoint of bronze passed into the bone", as well as mentions on the loss of conscious post-neurotrauma in the poetic description "and darkness enfolded his eyes, and he crashed as does a wall, in the mighty combat" [2]. During this period, the first instance of concussion leading to changes in personality was mentioned in the works of Plutarch with regards to the accident suffered by Aridaios [2].

The Hippocratic period is named after Hippocrates of Kos, who is considered the "father of Medicine" and contributed tremendously to the advancement of medicine and knowledge on neurotrauma by performing the first systematic approach to TBI in his work: "On injuries of the head" [5]. In his treatise, he pinpoints various surgical procedures for different skull fractures and brain injuries, mentions the clinical applications of trepanation, recognizes the implications of infections post-trepanation, proposes a new classification of fractures (fissured fractures, bone contusions, depressed skull fractures, contrecoup fractures and fractures of the cranial sutures), and introduces the importance of patient records in cases of TBI [5]. Hippocrates carried out detailed observations of cranial anatomy through clinical examination rather than dissections. He concentrated his efforts on reaching an accurate prognosis rather than proceeding with direct surgical interventions and tested for skull fractures using a metal probe [5, 7]. In addition, he supported the use of trepanation for fractures, contusions, and head injuries, associated with bone contusion, referencing and describing the surgical technique [8]. One interesting case presented in his fifth book of Epidemics details the management of a head injury in an 11-year-old boy who suffered a kick to the forehead caused by a horse [7]. The "father of medicine" opposed the principles of magic and superstition, building a medical approach based on systematic observation, thus becoming a true pioneer of TBI research [5].

Hippocrates (460–377 BC) wrote the memorable treatise "On Wounds in the Head", a valuable source of insight into head injuries in classical antiquity and the first work in history focusing exclusively on cranial trauma [9]. In his work, he offers helpful descriptions of the appearance and consistency of the cranium, explains pathophysiological processes, analyses clinical cases, classifies fractures, reveals mechanisms, clinical assessment, and empirical treatments, and illustrates the trepanation procedure [9]. His work represents true innovation, as it describes accurate anatomical observations noticed through surgical interventions, despite the lack of vivisections and dissections in that period [1, 9].

Another bright mind of the time, Plato (429–347 BC), regarded the brain as the "cranial marrow" and the spinal cord as the "vertebral marrow" [1]. Aristotle (384–322 BC) illustrated the concept by comparing the existence of the spinal cord, which he described as "vertebral marrow" ("spondylion") to the presence of marrow found in bones [1]. Hippocratic writers later understood that injury to the spinal cord could cause paralysis. There are even passages in the Iliad that suggest a possible relationship between spinal cord injury and paralysis, as well as an understanding of the role of the brachial plexus, illustrated by descriptive passages "The nerves broke, and the hand became asleep unto the wrist" [1].

Other notable contributions in the domain of neuroscience were those of Praxagoras of Kos, who suggested the existence of neurons [10]. Furthermore, Herophilus of Chalcedon made the distinctions between sensory and motor nerves, showed the presence of the nervous system through dissection, and made the distinction between the cerebrum and cerebellum. Finally, Alcmaeon postulated that "the brain creates the mind" and is connected to the body through channels while also suggesting the brain to be the nucleus of Perception [10].

The story of neurotrauma in Ancient Greece is fascinating, as it highlights the precocity of great minds in understanding processes that would later be defined by modern medicine. Furthermore, the detailed and vivid observations of poets and writers stand as a testament that medicine and science can intertwine with art and that humanity is only going to benefit from multidisciplinary approaches to advance knowledge on neurotrauma and any other domain.
